# Isolated para-aortic lymph node recurrence from colorectal cancer treated by radiotherapy: a systematic review and meta-analysis

**DOI:** 10.1038/s41598-026-41478-3

**Published:** 2026-03-04

**Authors:** Seok-Joo Chun, Hyunkyung Kim, Jiyun Jung, Sun Hyun Bae, Mi-Sook Kim

**Affiliations:** 1 Department of Radiation Oncology, Ilsan Dongguk University Hospital, Goyang, Republic of Korea; 2 Department of Radiation Oncology, Korea Institute of Radiological & Medical Sciences, Seoul, Republic of Korea; 3 Department of Biostatistics, Dongguk University College of Medicine, Gyeongju, Republic of Korea; 4 Department of Radiation Oncology, Soonchunhyang University College of Medicine, Bucheon, Republic of Korea

**Keywords:** Radiotherapy, Oligometastasis, Para-aortic, Colorectal neoplasm, Cancer, Medical research, Oncology

## Abstract

Optimal management for isolated para-aortic lymph node (PALN) recurrence from colorectal cancer (CRC) remains undefined. We evaluated the efficacy and safety of radiotherapy (RT) for this indication. A PRISMA-compliant systematic review and meta-analysis (PROSPERO CRD42025634494) was conducted using four databases through April 2025. Pooled locoregional progression-free survival (LPFS), progression-free survival (PFS), and overall survival (OS) were estimated using a random-effects model. Five retrospective studies (*n* = 220) were included. Pooled 1- and 3-year LPFS were 84% (95% CI, 78–89%) and 69% (62–76%), respectively. Pooled 1- and 3-year PFS were 54% (42–65%) and 22% (14–32%). Pooled 1- and 3-year OS were 91% (87–95%) and 64% (54–73%). Primary colon tumors were associated with poorer survival outcomes, while female sex predicted improved 3-year PFS. Severe (grade ≥ 3) acute and chronic toxicities were low (7.7% and 0.5%, respectively). Modern RT for PALN recurrence from CRC provides favorable local control with acceptable toxicity. With a 3-year OS of 64%, RT offers a potential survival benefit for carefully selected patients.

**Supplementary Information:**

The online version contains supplementary material available at 10.1038/s41598-026-41478-3.

## Introduction

Colorectal cancer is one of the most frequently diagnosed malignancies worldwide, ranking as the third most common cancer and the second leading cause of cancer-related mortality^1,2^. Surgical resection remains the cornerstone of curative treatment, with perioperative chemotherapy and radiotherapy recommended for patients at high risk of recurrence, such as those with advanced stage disease, lymphovascular invasion, or inadequate nodal harvest^3,4^. Despite advances in surgical techniques, perioperative management, and systemic therapy, a substantial proportion of patients—reported in up to 30–50%—experience disease recurrence during follow-up^5–7^.

The most common sites of recurrence are the liver, lungs, and locoregional lymph nodes, reflecting the characteristic metastatic spread patterns of colorectal cancer^8,9^. Para-aortic lymph node (PALN) recurrence, while relatively rare compared to hepatic or pulmonary metastases, represents a distinct pattern of locoregional–distant spread^10^. The reported incidence of PALN recurrence ranges from approximately 1% to 2% following curative resection, and it is more frequently observed in patients with advanced nodal disease or tumors involving the sigmoid colon and rectum^11^. Although uncommon, PALN recurrence is clinically important due to its association with poor prognosis and the absence of standardized management guidelines.

Treatment strategies for isolated PALN recurrence vary widely and include surgical resection, radiotherapy, systemic chemotherapy, and multimodality approaches, with selection guided by disease extent, patient performance status, and institutional expertise^12–19^. In the broader context of oligometastatic disease—characterized by a limited metastatic burden amenable to curative-intent local therapy—radiotherapy has accumulated supportive evidence, particularly with stereotactic body radiotherapy (SBRT) and highly conformal techniques^20–27^. Modern image guidance and intensity-modulated or volumetric techniques enable delivery of ablative doses while respecting adjacent organs at risk (e.g., bowel, duodenum, kidneys, spinal cord), and observational studies suggest meaningful local control with acceptable toxicity for isolated nodal metastases, including PALN involvement^28–30^. Nevertheless, the evidence base remains constrained by small sample sizes, retrospective designs, and heterogeneity in patient selection, dose/fractionation, and concurrent systemic therapy. Given the paucity of well-designed studies specifically addressing isolated PALN recurrence from colorectal cancer, we aimed to evaluate the clinical outcomes of radiotherapy by conducting a systematic review and meta-analysis.

## Materials and methods

### Study protocol and Search strategy

A systematic review was conducted according to Preferred Reporting Items for Systematic Review and Meta-Analysis (PRISMA) statement^31^. The protocol was specified a priori and registered in PROSPERO (CRD42025634494). We systematically searched MEDLINE (PubMed), Embase, Web of Science, and the Cochrane Library from database inception to April 1, 2025, without initial date limits. Search strings combined controlled vocabulary and keywords for the anatomic region, disease, and intervention. A representative strategy was (para-aortic OR paraaortic OR retroperitoneal OR aortic OR aortocaval) AND (colorectal OR colon OR rectal) AND (radiotherapy OR RT OR radiation). Reference lists of eligible studies and relevant reviews were hand-searched to identify additional reports. No language limits were applied at the search stage; only English-language full texts were included at screening. Two reviewers (S.J.C. and H.K.K.) independently screened records and assessed full texts; disagreements were resolved through discussion and, when necessary, adjudicated by a third reviewer (M.S.K.).

### Eligibility criteria

Studies were eligible if they met all of the following criteria: (1) adults with histologically confirmed colorectal cancer who had undergone curative-intent resection of the primary tumor; (2) metachronous, isolated PALN recurrence diagnosed radiologically and/or pathologically; (3) delivery of radiotherapy to PALN disease using modern technique such as 3D-conformal or intensity-modulated radiotherapy; (4) reporting at least one prespecified clinical outcome — including local progression-free survival (LPFS), progression-free survival (PFS), overall survival (OS), objective response rate, or acute and/or late toxicity; and (5) retrospective or prospective clinical study design. We excluded the following: (1) studies involving pediatric populations; (2) radiotherapy administered in the perioperative setting; (3) non-clinical or technical reports (e.g., dosimetric or planning-only studies) and conference abstracts without extractable outcomes; (4) case reports or very small series (< 5 patients); and (5) non-English full-text articles when translation was not available.

### Data extraction and Quality assessment

Two authors (S.J.C. and H.K.K.) independently extracted data from all eligible studies. Extracted variables included patient characteristics (age, sex, and performance status) and tumor-related factors such as the initial cancer site, interval from primary surgery to recurrence, and initial stage. Radiotherapy-related parameters were also collected, including gross tumor volume (GTV), total radiation dose, number of fractions, and the equivalent dose in 2 Gy fractions (EQD2). EQD2 was calculated using the linear–quadratic model with an α/β ratio of 10 Gy. Survival outcomes were collected for 1-year and 3-year LPFS, PFS, and OS. Reported toxicities were extracted as described in the original articles, with grade ≥ 3 events according to the Common Terminology Criteria for Adverse Events (CTCAE) considered severe. Acute toxicities were defined as those occurring during radiotherapy or within 3 months after its completion, whereas chronic toxicities were defined as those manifesting beyond 3 months post-treatment.

As all included studies were retrospective in nature, the methodological quality of each study was assessed using the Newcastle–Ottawa Scale (NOS), which is specifically designed for evaluating nonrandomized studies in meta-analyses^32^. Two reviewers performed the assessment independently, and any discrepancies were resolved through discussion and consensus.

### Statistical analysis

We conducted a meta-analysis of the probability for LPFS, PFS, and OS at 1 and 3 years. To stabilize variance and satisfy the assumption of normality, proportions were estimated using log transformation^33^. Pooled effect sizes were calculated with the inverse variance method under a random-effects model, which was applied to account for between-study heterogeneity encountered in various studies. The heterogeneity was presented as I² (25%: low, 50%: moderate; >75% high) and p-value for Cochran’s Q (p-value < 0.05) that indicates whether the observed variation is due to actual differences rather than chance^34^. In addition, the τ² statistic, with higher values indicating greater heterogeneity across studies, was used to estimate the between-study variance^34^.

Univariable random-effects meta-regression was also performed to explore potential factors, including age, sex, initial site (colon vs. rectal), and EQD2, associated with the probability of LPFS, PFS, and OS. All analyses were conducted using the *metafor* packages on R software (version 4.1.1; R Core Team, Vienna, Austria) and a p-value under 0.05 considered statistically significant.

## Results

**Fig. 1:**
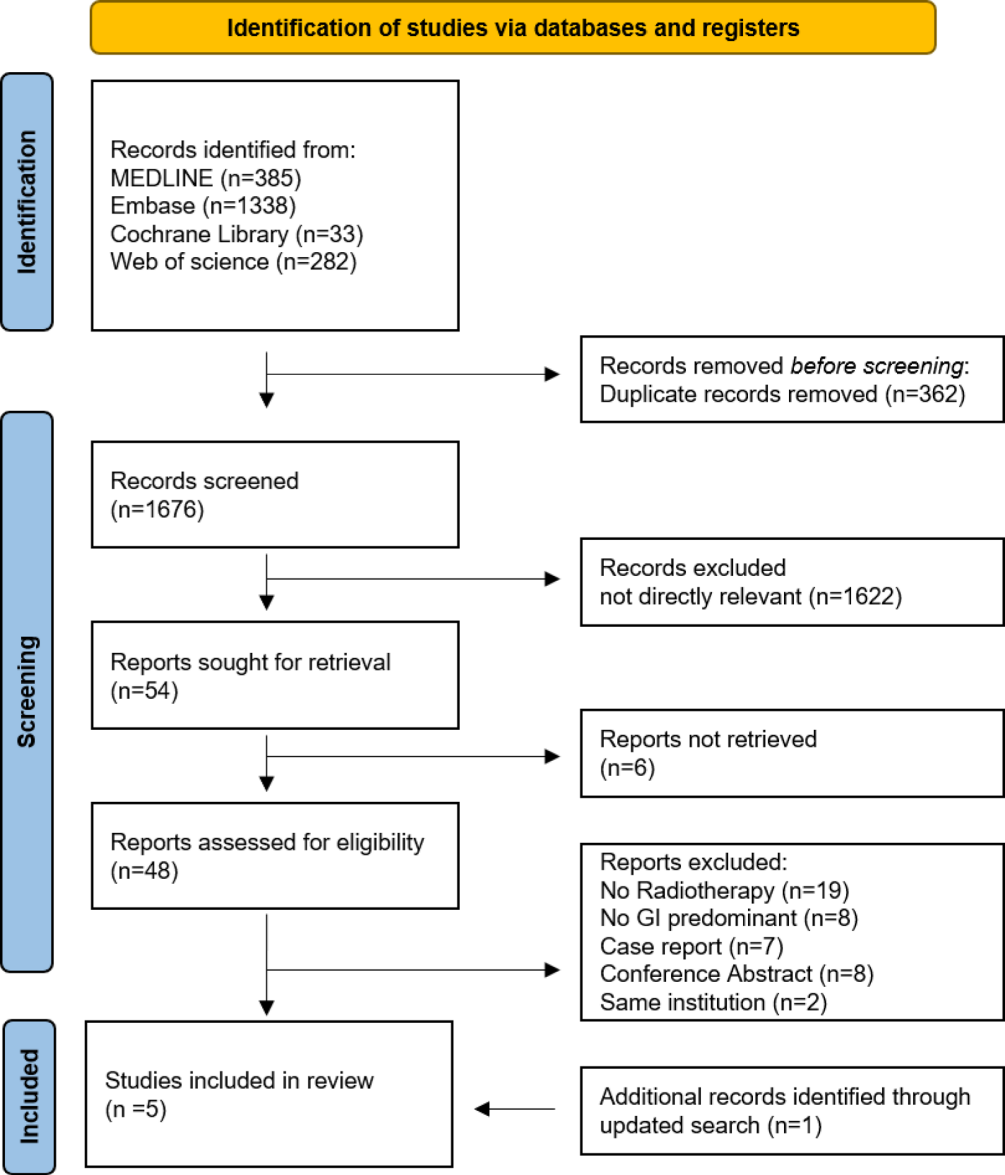
PRISMA flowchart for the meta-analysis of para-aortic lymph node metastasis from colorectal cancer.

From the initial database search, a total of 2,038 articles were identified, including 385 from MEDLINE, 1,338 from Embase, 33 from the Cochrane Library, and 282 from Web of Science. After removal of duplicate records, 1,676 unique articles remained for screening. Title and abstract screening identified 48 studies as potentially relevant to this review. After full-text assessment, 6 studies met all eligibility criteria. However, we identified a newly published article which incorporated two studies that were already included in our review, and these duplicates were excluded. In total, the meta-analysis included 5 independent studies, from which 6 effect sizes were extracted. These selected studies specifically addressed the treatment outcomes of radiotherapy for isolated PALN recurrences in colorectal cancer. Figure 1 represents the PRISMA flowchart of meta-analysis.

All five studies included in this systematic review were retrospective in nature, encompassing a total of 220 patients diagnosed with isolated PALN recurrence from colorectal cancer^15,28,32,35,36^. The studies were conducted across multiple countries, including Korea, Italy, Japan, the United States, and China, with study periods ranging from 2002 to 2023. Sample sizes varied considerably, from as few as 7 patients to as many as 116. Median ages ranged between 54 and 66 with a range of 23 to 89. Study quality, assessed using the NOS, ranged from 5 to 7, indicating moderate to high quality. All studies involved patient cohorts in which all participants received radiotherapy, either as monotherapy or in combination with chemotherapy. A detailed summary of study characteristics is provided in Table 1, and the chemotherapy regimens used across the studies are summarized in *Supplementary Table 1*.

**Table 1:** Eligible studies for radiotherapy to metachronous para-aortic lymph node metastasis from colorectal cancer.

Author (year)	Country	Study type	Study period	Sample size	Age (years)	Sex (F/M)	Initial tumor (Col vs. Rec)	Initial T Staging (T1,2 vs. T3,4)	Initial *N* Staging (N0 vs. *N*+)	Initial Staging (I, II vs. III/IV)	Time from surgery to recurrence (months)	CTx	NOS
Kim (2009)[32]	Korea	R/SC	2003–2009	7	59 (47–73)	2/5	0/7	n/a	n/a	2/5	21 (7–44)	All before SBRT	5
Yeo (2010) [25]	Korea	R/SC	2002–2007	22	54 (27–70)	11/11	9/13	2/20	3/19	2/20	15 (1–70)	All CCRT 16 Adj CTx	6
Franzese (2017)[33]	Italy	R/SC	2008–2015	35	66 (32–89)	11/24	9/24	n/a	n/a	n/a	45 (7–86)	n/a (no CCRT allowed)	5
Shu (2020)[14]	China	R/SC	2009–2018	40	58 (30–78)	19/21	8/32	n/a	n/a	8/28	n/a	All before RT (7 CCRT)	6
Lee (2025)[29]	Korea/Japan	R/MC	2006–2023	53 (Carbon)	63 (31–85)	24/29	29/24	7/35	10/32	n/a	n/a	21 before RT	7
				63 (Photon)	58 (23–86)	22/41	35/28	10/53	14/49	n/a	n/a	44 CTx (6 CCRT)	

Abbreviations: Adj, adjuvant; CCRT, concurrent chemoradiotherapy; Col, colon; CTx, chemotherapy; ECOG, Eastern Cooperative Oncology Group; MC, multicenter; n/a, not available; NOS, Newcastle-Ottawa scale; PAN, para-aortic nodes; PS, performance status; R, retrospective; Rec, rectal; RT, radiotherapy; SBRT, stereotactic body radiotherapy; SC, single center.

The median follow-up duration across the included studies was 36 months, ranging from 15 to 56 months. The median prescribed radiotherapy dose was 54.5 Gy (range, 45–63 Gy), delivered in various fractionation schemes. The equivalent dose in 2 Gy fractions (EQD2, α/β = 10) ranged from 57.12 Gy to 104.0 Gy, with a median of 62.66 Gy. Treatment outcomes varied among studies but generally demonstrated favorable local control. Reported 1-year LPFS ranged from 85.3% to 100.0%, and 3-year LPFS ranged from 61.1% to 85.7%. PFS was more variable, with 1-year rates between 38.5% and 69.7%, and 3-year rates between 10.0% and 34.1%. OS was relatively high in several reports, with 1-year OS ranging from 89.8% to 100.0% and 3-year OS from 42.9% to 81.4%. Treatment profile and treatment outcome are summarized in Table 2.

**Table 2:** Treatment related factors and treatment outcome for included studies.

Author (year)	Treatment	Study F/U (mo/ m)	GTV size (cc)	RT dose (Gy)	RT fraction	EQD2 (Gy)	1-year LPFS (%)	3-year LPFS (%)	1-year PFS (%)	3-year PFS (%)	1-year OS (%)	3-year OS (%)
Kim (2009)	Photon (SBRT)	26 (21–70)	22 (4–40)	48 (36–51)	3 (3–3)	104	100	85.7	57.1	14.3	100	42.9
Yeo (2010)	Photon	34 (29–63)	2* (0.7–3.3)	63 (56–63)	35 (31–35)	62.0	n/a	76.4	69.7	34.1	95.4	64.7
Franzese (2017)	Photon	15 (2–68)	8 (0.2–568)	45 (30–45)	6 (5–13)	65.6	85.3	75.0	69.4	19.4	100	81.4
Shu (2020)	Photon	38 (5-119)	n/a	60 (40–66)	n/a	60.0	87.5	70.0	57.9	31.3	89.8	75.4
Lee (2025)	Carbon	41 (4-139)	5.4 (1-584)	53 (48–55)	12	63.4	86.5	72.2	41.2	18.4	91.1	59.3
	Photon	56 (10–156)	11 (1-101)	56 (25–65)	25	57.1	77.8	61.1	38.5	10.0	91.1	53.6

Abbreviations: EQD2, equivalent dose in 2 Gy fractions; F/U, follow-up; GTV, gross tumor volume; LPFS, local progression-free survival; n/a, not available; OS, overall survival; PFS, progression-free survival; RT, radiotherapy; SBRT, stereotactic body radiotherapy; *: Reported tumor size **: Median EQD2 with alpha/beta ratio of 10.

**Fig. 2:**
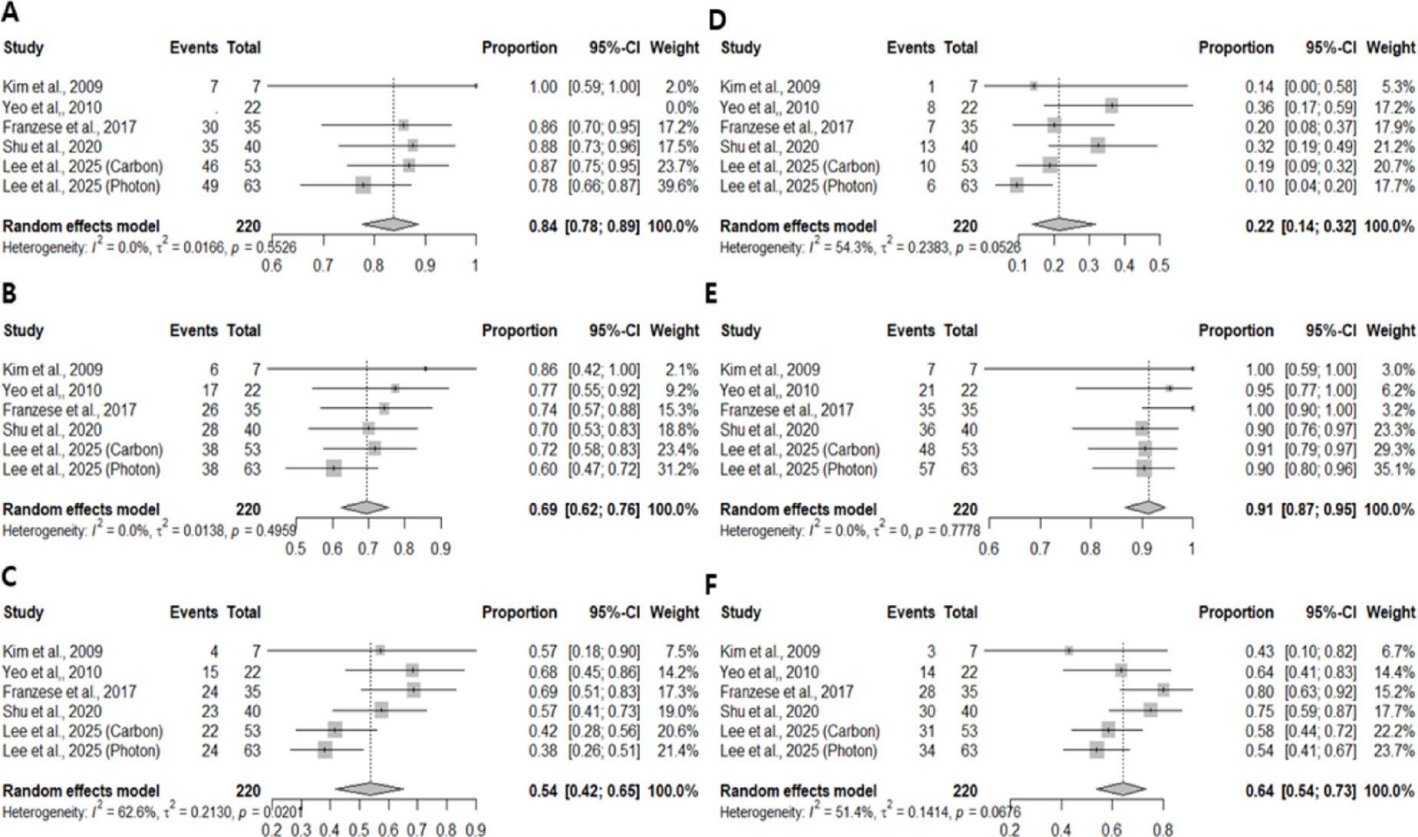
Forrest plot of 1-year LPFS (**A**), 3-year LPFS (**B**), 1-year PFS (**C**), 3-year PFS (**D**), 1-year OS (**E**), and 3-year OS (**F**) of total patients.

Pooled analyses were conducted using a random-effects model. The estimated 1-year and 3-year LPFS rates were 84% (95% CI, 78–89%) and 69% (95% CI, 62–76%), respectively, with low heterogeneity (I² = 2.0% and 0.0%). For PFS, the pooled 1-year and 3-year rates were 54% (95% CI, 42–65%) and 22% (95% CI, 14–32%), respectively. The pooled 1-year and 3-year OS rates were 91% (95% CI, 87–95%) and 64% (95% CI, 54–73%), respectively. Figure 2 presents the forest plots for LPFS, PFS, and OS.

We have performed univariable meta-regression for factors that are associated with LPFS, PFS and OS (Table 3). Primary colon tumors (vs. rectal) were significantly associated with poorer PFS (coefficient − 2.37, 95% CI − 4.01 to − 0.73, *p* < 0.01) and poorer 3-year OS (coefficient − 1.83, 95% CI − 3.55 to − 0.11, *p* = 0.04). Female was associated with improved 3-year PFS (coefficient 6.27, 95% CI 1.39–11.15, *p* = 0.01). Nodal status (N0) at initial diagnosis had effects on reduced PFS (–14.56, 95% CI − 27.04 to − 2.09, *p* = 0.02) and OS (–20.81, 95% CI − 35.53 to − 6.08, *p* = 0.01). Similarly, lower initial T stage (T1–2) compared to higher stage (T3–4) was associated with poorer PFS (–15.88, 95% CI − 29.86 to − 1.90, *p* = 0.03) and OS (–24.08, 95% CI − 41.18 to − 6.98, *p* = 0.01).

**Table 3:** Meta-regression analysis of factors related with para-aortic lymph node metastasis from colorectal cancer.

Factors	LPFS				PFS					OS		
	1-year		3-year		1-year		3-year		1-year		3-year	
	Coefficient	p-value	Coefficient	p-value	Coefficient	p-value	Coefficient	p-value	Coefficient	p-value	Coefficient	p-value
	(95% CI)		(95% CI)		(95% CI)		(95% CI)		(95% CI)		(95% CI)	
Age*	0.04	0.49	0.02	0.59	0	0.96	-0.05	0.42	0.07	0.37	0.05	0.35
	(-0.08,0.17)		(-0.06,0.10)		(-0.11,0.12)		(-0.17,0.07)		(-0.08,0.21)		(-0.05,0.15)	
Sex	1.75	0.55	1.08	0.6	0.31	0.92	**6.27**	**0.01**	-4.05	0.28	0.68	0.8
(ref. Male)	(-3.94,7.44)		(-2.93,5.08)		(-5.45,6.06)		**(1.39**,**11.15)**		(-11.46,3.36)		(-4.59,5.95)	
Initial tumor	-1.61	0.18	-1.15	0.2	**-2.37**	**< 0.01**	-1.74	0.14	-1.96	0.26	**-1.83**	**0.04**
(ref. Rectum)	(-3.97,0.74)		(-2.92,0.62)		**(-4.01**,**-0.73)**		(-4.04,0.57)		(-5.38,1.46)		**(-3.55**,**-0.11)**	
Initial staging	n/a		-0.13	0.98	-3.07	0.44	-3.67	0.37	-1.06	0.89	-0.85 (-8.86,7.17)	0.84
(ref. Stage III/IV vs. I/II)			(-8.62,8.36)		(-10.80,4.66)		(-11.75,4.41)		(-15.49,13.36)			
Initial T staging	n/a		-11.06	0.12	**-14.56**	**0.02**	**-20.81**	**0.01**	-7.94	0.51	-5.12 (-17.43,7.19)	0.42
(ref. T3/4 vs. T1/2)			(-24.95,2.82)		**(-27.04**,**-2.09)**		**(-35.53**,**-6.08)**		(-31.48,15.61)			
Initial N staging	n/a		-12.62	0.11	**-15.88**	**0.03**	**-24.08**	**0.01**	-8.36	0.53	-5.78 (-19.61,8.04)	0.41
(ref. N + vs. N0)			(-28.05,2.81)		**(-29.86**,**-1.90)**		**(-41.18**,**-6.98)**		(-34.22,17.49)			
Time from surgery to recurrence*	n/a		-0.01	0.68	0	0.85	-0.02	0.25	n/a		0.03	0.09
			(-0.05,0.03)		(-0.03,0.04)		(-0.06,0.02)				(-0.01,0.07)	
EQD2*	0.08	0.14	0.04	0.16	0.01	0.59	-0.01	0.82	0.12	0.16	-0.01	0.48
	(-0.03,0.19)		(-0.01,0.09)		(-0.03,0.05)		(-0.06,0.04)		(-0.05,0.28)		(-0.05,0.03)	

Abbreviation: EQD2, equivalent dose in 2 Gy fractions; LPFS, Local progression-free survival; PFS, progression-free survival; OS, overall survival. *: Estimated as continuous variables. A positive coefficient indicates a better treatment outcome compared with the reference category, whereas a negative coefficient indicates a poorer outcome. Bold values denote statistical significance (*p* < 0.05).

Radiotherapy was generally well tolerated across the included studies, with severe toxicities (grade ≥ 3) occurring in a total of 18 patients (8.2%). Acute severe toxicities were observed in 17 patients (7.7%), including 12 hematopoietic, 3 gastrointestinal, and 2 hepatic events. One patient (0.5%) experienced late toxicity, a case of grade 4 colonic obstruction. A detailed summary of toxicity profiles from each study is presented in Table 4.

**Table 4:** Toxicity profile for radiotherapy to para-aortic lymph node metastasis.

Author (year)	Sample size	Treatment	Study F/U (mo/ m)	Toxicity Criteria	Number of Toxicity	Acute G3 + toxicity	Chronic G3 + toxicity
Kim (2009)	7	Photon (SBRT)	26 (21–70)	Gr 1 Nausea/Vomiting Gr 4 Colon obstruction	2 (28.6%) 1 (14.3%)	0	1 (14.3%)
Yeo (2010)	22	Photon	34 (29–63)	Gr 1–2 GI toxicity	18 (81.8%)	0	0
Franzese (2017)	35	Photon	15 (2–68)	Gr 1 Nausea Gr 2 Diarrhea, Nausea	2 (5.7%) 3 (8.6%)	0	0
Shu (2020)	40	Photon	38 (5-119)	Gr 1 GI toxicity Gr 3 Hematologic Gr 3 Liver injury Gr 3 GI toxicity	5 (12.5%) 3 (7.5%) 2 (5.0%) 1 (2.5%)	6 (15.0%)	0
Lee (2025)	53	Carbon	41 (4-139)	Gr 2 Hematopoietic Gr 3 Hematopoietic	7 (13.2%) 5 (9.4%)	5 (9.4%)	0
	63	Photon	56 (10–156)	Gr 2 GI toxicity Gr 2 Hematopoietic Gr 3 GI toxicity Gr 3 Hematopoietic	9 (14.3%) 7 (11.1%) 2 (3.2%) 4 (6.3%)	6 (9.5%)	0

Abbreviation: F/U, follow-up; G3+, grade 3 or more; GI, gastrointestinal; Gr, grade; RT, radiotherapy; SBRT, stereotactic body radiotherapy.

## Discussion

To our knowledge, this study represents the first meta-analysis to systematically evaluate the clinical outcomes of radiotherapy for isolated PALN recurrence originating from colorectal cancer. Our pooled analysis, encompassing five retrospective studies with a total of 220 patients, demonstrated favorable rates of local disease control, with a 1-year LPFS of 84% and a 3-year rate of 69%. Furthermore, radiotherapy was well tolerated, with severe acute and chronic toxicities observed in 7.7% and 0.5% of patients, respectively.

The management of oligometastatic colorectal cancer has increasingly incorporated aggressive local therapies for sites such as the liver and lungs, where randomized and observational studies have demonstrated survival advantages compared with systemic therapy alone^5,6,37–40^. Given the relatively indolent disease course in selected patients, local approaches in the oligometastatic setting have been extensively studied and are now widely adopted. Extending this paradigm to PALN recurrence, surgical resection has traditionally been regarded as a potentially curative strategy, with select series reporting long-term survival in carefully chosen patients^17,30,41–44^. Zizzo et al. conducted a systematic review on surgical resection for isolated PALN recurrences, reporting 3-year and 5-year OS rates ranging from 59.4% to 68.0% and from 53.4% to 87.5%, respectively^17^. Ito et al. reported a 5-year recurrence-free survival rate of 21.1%, with a median recurrence-free survival of 1.2 years, in a multicenter retrospective study involving 133 patients^43^. Nonetheless, this approach presents considerable technical challenges. The para-aortic region lies deep within the retroperitoneum and in close proximity to major vascular and visceral structures—including the great vessels, renal vasculature, and duodenum—making surgical dissection technically demanding and associated with non-negligible morbidity^45^. Reported perioperative complication rates range from approximately 15% to nearly 40% in contemporary series^17,41,43^.

In this context, radiotherapy may represent a feasible non-invasive alternative that can effectively overcome the anatomical barriers limiting surgery. Our meta-analysis suggests that radiotherapy achieves survival outcomes comparable to those reported in surgical series, with a pooled 3-year OS of 64% compared with published rates of 59.4–68.0% for surgery^17,30^. Moreover, the safety profile of modern radiotherapy for PALN lesions appears compelling. The low rates of severe toxicities observed in our analysis (7.7% acute, 0.5% chronic) compare favorably with the 15–40% perioperative complication rates reported for surgical resection.

However, this indirect comparison must be interpreted with caution due to inherent selection bias between potentially distinct patient cohorts. This limitation underscores a broader theme: the challenge of balancing aggressive local treatment against the high risk of systemic failure. This dilemma is further illustrated by outcomes from intensive trimodality regimens. While such approaches achieve excellent local control, the corresponding 3-year PFS of only 36% demonstrates that a large proportion of patients subsequently develop systemic disease, raising questions about the risk-benefit balance of maximizing local therapy intensity^16^. Accordingly, radiotherapy may offer a more balanced therapeutic approach, achieving durable local control with lower morbidity while preserving the opportunity for timely systemic treatment.

The challenge of systemic control naturally leads to the consideration of chemotherapy; however, its optimal integration with local treatment in the PALN recurrence setting is still evolving^46^. Most patients in the studies included in this meta-analysis received chemotherapy in combination with radiotherapy, either concurrently or sequentially^15,28,32,35,36^. This approach combines chemotherapy to target micrometastatic disease with radiotherapy for durable local control, a strategy proven effective in other colorectal oligometastatic sites like the liver and lung^47–52^. Nevertheless, the precise benefit of this integration remains difficult to quantify due to heterogeneous regimens and retrospective study designs. Therefore, a critical unmet need is the prospective evaluation of combination strategies to define optimal sequencing and chemotherapy regimens.

Beyond the integration of systemic therapy, technical aspects of radiotherapy itself warrant discussion. Across the included studies, prescribed doses and EQD2 values were relatively uniform, limiting conclusions regarding the benefit of dose escalation. One small SBRT series reported excellent outcomes, with 1- and 3-year LPFS rates of 100% and 85.7%, respectively^35^. In our pooled analysis, higher EQD2 showed a non-significant trend toward improved local control, serving as a hypothesis-generating signal for the potential role of dose intensification. Beyond photon therapy, particle therapy has emerged as a promising alternative. Retrospective comparative data between photon and carbon-ion radiotherapy demonstrated superior local control with carbon-ion treatment^32^, highlighting its potential advantages in overcoming anatomical limitations of dose delivery. Future research should prospectively evaluate optimal modality, dose-fractionation, and field-design strategies, while establishing multicenter registries to generate high-quality evidence and inform consensus guidelines for the management of isolated PALN recurrence.

Our meta-regression analysis identified several clinicopathologic variables with potential prognostic relevance. Notably, significant observation was the association of female sex with improved 3-year PFS, a finding consistent with prior evidence suggesting sex-related differences in colorectal cancer biology, immune modulation, and treatment response^53,54^. Another observation was that the patients with colon primary tumors exhibited poorer PFS and OS compared to those with rectal primaries. This finding contrasts with the conventional understanding that rectal cancers typically demonstrate more aggressive locoregional behavior and poorer prognosis compared to colon cancers^55,56^. Furthermore, the observation that patients with lower initial T or N stage experienced worse outcomes following PALN recurrence is paradoxical. This divergence from the general understanding may not be conclusive due to the limited number of cohorts, and such variables would not be regarded as selection factors for optimal candidates for radiotherapy or other local treatment modalities.

This study has several limitations. The number of eligible studies and patients was small, reflecting the rarity of isolated PALN recurrence, and all included reports were retrospective, limiting statistical power and raising the possibility of selection bias. Furthermore, as the included studies span more than two decades (2002–2023), various radiotherapy techniques were employed, which might have influenced the consistency of treatment outcomes. Additionally, variations in target volume definitions—ranging from GTV-based focal treatment to elective nodal irradiation—were observed across the cohorts; however, the limited number of studies precluded further subgroup analysis to evaluate their respective impacts on efficacy and toxicity. Lastly, the limited number of available studies precluded detailed comparison among surgery, radiotherapy, and chemotherapy, making it difficult to delineate the relative role of each treatment modality in the management of PALN recurrence.

In conclusion, we conducted the first systematic review and meta-analysis evaluating radiotherapy for isolated PALN recurrence from colorectal cancer. Despite inherent patient heterogeneity, this meta-analysis suggests that modern radiotherapy for PALN recurrence from colorectal cancer yields favorable local control and acceptable toxicity. With 3-year overall survival rates exceeding 60% and progression-free survival in approximately one-fifth of patients, these findings indicate that radiotherapy may offer a potential survival benefit for carefully selected patients.

## Supplementary Information

Below is the link to the electronic supplementary material.

Supplementary Material 1

Supplementary Material 2

## Data Availability

All data analyzed during this study were extracted from previously published articles and are publicly available in the cited references. No individual patient data were generated. The compiled dataset used for analysis is available from the corresponding author upon reasonable request.
