# Supplementary material for: Isolated para-aortic lymph node recurrence from colorectal cancer treated by radiotherapy: a systematic review and meta-analysis
Source: Sci Rep. 2026 Mar 4;16:11980. doi: 10.1038/s41598-026-41478-3 (PMC13069108; doi:10.1038/s41598-026-41478-3)
Supplement: Supplementary file 1 — Supplementary Material 1 [file 41598_2026_41478_MOESM1_ESM.docx]

## **Supplementary Table 1.** Radiotherapy field and chemotherapy regimen used in each study

| Author  (year) | Sample Size | Radiotherapy Modality | Radiotherapy  Field | Chemotherapy regimen |
| --- | --- | --- | --- | --- |
| Kim (2009) | 7 | Photon (SBRT) | GTV(=CTV) + 2-3mm | All 5-FU based CTx before RT |
| Yeo (2010) | 22 | Photon | GTV + elective LNs | All received CCRT  4 FL  14 Capecitabine alone  2 CAPOX  2 Capecitabine plus Irinotecan |
| Franzese (2017) | 35 | Photon | GTV(=CTV) + 5mm | n/a (CCRT not allowed) |
| Shu (2020) | 40 | Photon | GTV + 2-3cm (CTV)  + 0.5cm (PTV) | All CTx before RT  20 FOLFIRI  9 FOLFOX6  11 CAPOX |
| Lee (2025) | 53 | Carbon | GTV + 1cm (CTV)  +0.2-0.8cm (PTV) | 21 before RT  8 FOLFOX  4 FOLFIRI  3 CAPOX  2 TS-1  4 Others |
|  | 63 | Photon | Similar principles applied in carbon treatment | 6 CCRT (4 FL, 2 capecitabine)  38 before RT  13 FOLFOX  22 FOLFIRI  3 Others |

Abbreviation: CAPOX, Capecitabine and Oxaliplatin; CCRT, concurrent chemoradiotherapy; CTx, chemotherapy; CTV, clinical target volume; FL, 5-FU and Leucovorin; FOLFIRI, 5-FU, Leucovorin and Irinotecan; FOLFOX, 5-FU, Leucovorin and Oxaliplatin; GTV, gross tumor volume; PTV, planning target volume; RT, radiotherapy; SBRT, stereotactic body radiotherapy
